# Forecasting the 2001 Foot-and-Mouth Disease Epidemic in the UK

**DOI:** 10.1007/s10393-017-1293-2

**Published:** 2017-12-13

**Authors:** David W. Shanafelt, Glyn Jones, Mauricio Lima, Charles Perrings, Gerardo Chowell

**Affiliations:** 1 Centre for Biodiversity, Theory and Modelling, Station d’Ecologie Théorique et Expérimentale du CNRS, Moulis, France; 2 Fera Science Ltd, Sand Hutton, York, UK; 3 Center of Applied Ecology and Sustainability (CAPES), Pontificia Universidad Católica de Chile, Casilla 114-D, 6513677 Santiago, Chile; 4 School of Life Sciences, Arizona State University, Tempe, AZ USA; 5 School of Public Health, Georgia State University, Atlanta, GA USA; 6 Division of International Epidemiology and Population Studies, Fogarty International Center, National Institutes of Health, Bethesda, MD USA

**Keywords:** Forecasting, Epidemic model, Foot-and-mouth disease, Sub-exponential growth

## Abstract

Near real-time epidemic forecasting approaches are needed to respond to the increasing number of infectious disease outbreaks. In this paper, we retrospectively assess the performance of simple phenomenological models that incorporate early sub-exponential growth dynamics to generate short-term forecasts of the 2001 foot-and-mouth disease epidemic in the UK. For this purpose, we employed the generalized-growth model (GGM) for pre-peak predictions and the generalized-Richards model (GRM) for post-peak predictions. The epidemic exhibits a growth-decelerating pattern as the relative growth rate declines inversely with time. The uncertainty of the parameter estimates $(r{\text{ and }}p)$ narrows down and becomes more precise using an increasing amount of data of the epidemic growth phase. Indeed, using only the first 10–15 days of the epidemic, the scaling of growth parameter (*p*) displays wide uncertainty with the confidence interval for *p* ranging from values ~ 0.5 to 1.0, indicating that less than 15 epidemic days of data are not sufficient to discriminate between sub-exponential (i.e., *p* < 1) and exponential growth dynamics (i.e., *p* = 1). By contrast, using 20, 25, or 30 days of epidemic data, it is possible to recover estimates of *p* around 0.6 and the confidence interval is substantially below the exponential growth regime. Local and national bans on the movement of livestock and a nationwide cull of infected and contiguous premises likely contributed to the decelerating trajectory of the epidemic. The GGM and GRM provided useful 10-day forecasts of the epidemic before and after the peak of the epidemic, respectively. Short-term forecasts improved as the model was calibrated with an increasing length of the epidemic growth phase. Phenomenological models incorporating generalized-growth dynamics are useful tools to generate short-term forecasts of epidemic growth in near real time, particularly in the context of limited epidemiological data as well as information about transmission mechanisms and the effects of control interventions.

## Introduction

Public health officials are increasingly recognizing the need to develop reliable disease forecasting approaches to respond to the increasing number of infectious disease outbreaks affecting the human population (Myers et al. 2000; Chretien et al. 2015). These epidemic forecasts consist of the stochastic ensemble of potential trajectories for an unfolding outbreak and can help guide the type and intensity of control strategies including vector control campaigns (Dinh et al. 2016) and healthcare infrastructure needs for diagnosis, isolation of infectious individuals, and contact tracing activities (Chretien et al. 2014; Viboud et al. 2016).

In this paper, we retrospectively assess the performance of simple phenomenological models that incorporate early sub-exponential growth dynamics for generating short-term forecasts of the 2001 foot-and-mouth disease (FMD) epidemic in the UK(Chowell et al. 2016a; Pell et al. 2016; Viboud et al. 2016). Specifically, we use the generalized-growth model (GGM) to (1) characterize the scaling of the epidemic growth phase through a deceleration of growth parameter (denoted by *p*) and (2) forecast the early growth dynamics of the epidemic. In addition, we employ the generalized-Richards model (GRM) to forecast the trajectory of the epidemic past the epidemic peak. It is worth noting that while several studies have used the daily series of notifications of infected premises during the 2001 foot-and-mouth disease epidemic in the UK to calibrate epidemic models (Kao 2002), few studies have used the data to test their capacity to forecast the trajectory of the epidemic (Morris et al. 2001). Rather, most existing studies use data on the whole epidemic to explain the driving factors of the epidemic or predict the effects of management policies. For example, Ferguson et al. (2001) and Keeling et al. (2001) reproduced the temporal dynamics of the 2001 epidemic to evaluate the performance of alternative management policies such as culling or vaccination. Later studies included the use of Markov Chain Monte Carlo methods to estimate epidemiological parameters of the 2001 epidemic for a number of transmission models (Chis Ster and Ferguson 2007) and mixed effects logistic regression methods to identify the risk factors for disease spread and predict which farms became infected (Bessell et al. 2010). We focus on the capacity of GGM and GRM models to predict the course of the epidemic from data made available as the epidemic progressed.

## Materials and Methods

### Background: The 2001 Foot-and-Mouth Disease Epidemic in the UK

**Figure 1:**
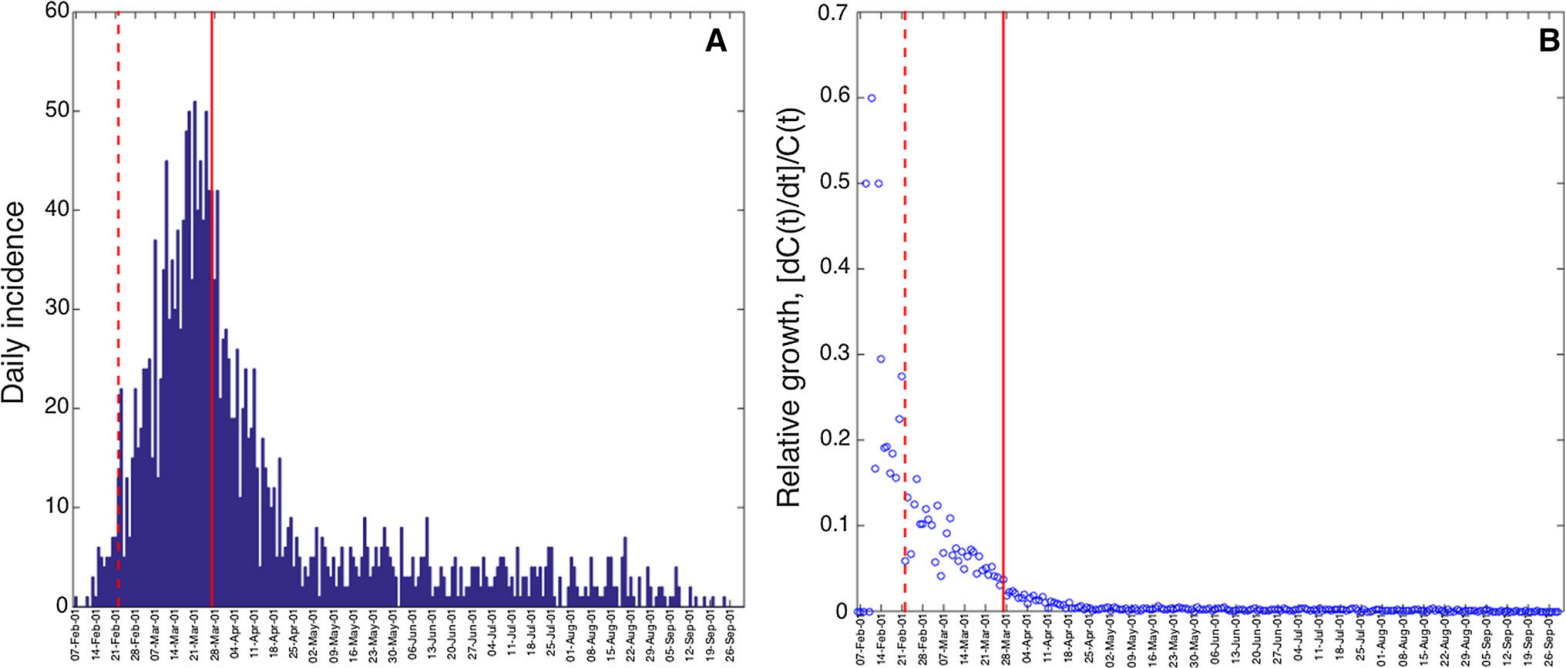
**a** Daily number of new notifications of infected premises during the 2001 foot-and-mouth disease epidemic in the UK and **b** the relative growth rate of the epidemic decreases inversely with time [see Arim et al. (2006)]. The vertical dashed line indicates the start of market closures on February 22, 2001 (16 days after the first notification), while the vertical solid line indicates the start of national culling policies on March 27, 2001 (49 days after the first notification), which occurred shortly after the epidemic peak.

The daily number of new, real-time notifications of infected premises during the 2001 foot-and-mouth disease epidemic in the UK was obtained from the Department of Environmental and Rural Affairs (DEFRA) database (DEFRA 2002) (Fig. 1). This epidemic resulted in the culling of over two million heads of livestock (Sobrino and Domingo 2001) and involved income losses to farmers, agriculture, the food chain, and tourist revenues estimated at £6.5 billion (Thompson et al. 2002). By the time the epidemic ended, 2026 farms had been infected making it the largest FMD epidemic in the UK (National Audit Office 2002). Additional 250 farms were suspected (not confirmed) as infectious, and another 8570 farms were culled preemptively (Tildesley et al. 2009). In total, almost ten percent of all farms in the country were culled due to FMD (Chis Ster and Ferguson 2007).

Several factors contributed to the size and scale of the epidemic: the undetected initial spread of the disease, heterogeneous farm biosecurity measures, and ineffective government incentives to mitigate the spread of disease (National Audit Office 2002). The virus initially spread via the transportation of undetected infected sheep throughout the country (Gibbens et al. 2001; Kao 2002; Kiss et al. 2006). This was partly because sheep show fewer physical symptoms of FMD than other susceptible species, making detection more difficult (Alexandersen et al. 2003). By the time the first case was confirmed, at least 57 farms were infected (National Audit Office 2002).^1^

The disease was first discovered in an abattoir (slaughterhouse) in Essex on February 20, 2001, and was traced back to a farm in Heddon-on-the-Wall in Northumberland 2 days later (National Audit Office 2002). After inspection, it was apparent that the premise had been infected for several weeks prior to detection (National Audit Office 2002). From the “case zero” premise in Heddon-on-the-Wall, the disease spread undetected locally and via the transportation of infected sheep to markets into Hexham, Carlisle, and Welshpool (Gibbens et al. 2001; National Audit Office 2002). Then, through livestock dealers, infected stock was transported across Great Britain. Consequently, bans on the local movement of livestock were imposed on February 21, 2001, while a national ban on the movement of livestock was initiated on February 23, 2001 (National Audit Office 2002).

The primary measures to control the epidemic included culling livestock on infected premises, preemptive culling on adjacent and/or “risky” premises, and culling to protect export markets (Anderson 2002; National Audit Office 2002; Tildesley et al. 2009; Woods 2013).^2^ In north Cumbria and southwest Scotland, local culls of at-risk premises and farms within a 3-km radius of an infected premise were implemented on March 22, 2001 (Tildesley et al. 2009). A nationwide preemptive cull of contiguous premises was approved on March 27, 2001 (National Audit Office 2002). In practice, however, preemptive culls were never fully implemented (National Audit Office 2002). While emergency vaccination was considered, it was never implemented (National Audit Office 2002). Epidemic control was most challenging in areas characterized by high degrees of initial seeding and in which farmers’ properties were distributed across several parcels of land hundreds of miles apart (National Audit Office 2002). It should be noted that in areas with few cases (e.g., < 10 outbreaks at a time) control was effective, reoccurrence of the disease once stamped out was rare, and bans on livestock movement kept the disease out of many of the profitable dairy and swine industries (National Audit Office 2002). Indeed, by January 22, 2002, Great Britain was reinstated as “disease-free, no vaccination” by the World Organization of Animal Health and by February 5, 2002, the European Commission lifted all remaining meat and animal export bans (National Audit Office 2002).

### Forecasting the National Trajectory of the Epidemic

#### The Generalized-Growth Model (GGM)

This simple phenomenological model relies on two parameters to characterize the early trajectory of an epidemic and to generate short-term epidemic forecasts (Chowell et al. 2016a; Pell et al. 2016; Viboud et al. 2016). The model incorporates epidemic growth patterns that range from sub-exponential (e.g., polynomial) to exponential by estimating two parameters: (1) the intrinsic growth rate (*r*) and (2) a dimensionless “deceleration of growth” parameter with quantified uncertainty (*p*). The latter modulates growth patterns ranging from constant incidence rates to exponential epidemic growth (Tolle 2003). It is useful to characterize the scaling of the growth pattern of the epidemic. In particular, this parameter is helpful to distinguish between sub-exponential ($p < 1$) and exponential growth dynamics ($p = 1$). Previous analyses highlighted the presence of early sub-exponential growth patterns in infectious disease data across a diversity of disease outbreaks including the 2013–2015 Ebola epidemic in West Africa, influenza, smallpox, plague, measles, foot-and-mouth disease, and HIV/AIDS (Viboud et al. 2016). The GGM model is given by the following differential equation (Tolle 2003; Viboud et al. 2016):

$$ \frac{{{\text{d}}C\left( t \right)}}{{{\text{d}}t}} = C^{{\prime }} \left( t \right) = rC\left( t \right)^{p} $$

where $C^{{\prime }} \left( t \right)$ describes the incidence curve over time $t$. The cumulative number of cases at time $t$ is given by $C\left( t \right)$, while $r$ is a positive parameter denoting the growth rate (1/time) and $p \in [ 0 , 1 ]$ is a “deceleration of growth” parameter. As described in Viboud et al. (2016), if $p = 0$, this equation describes a constant incidence over time, while if $p = 1$ the equation becomes the well-known exponential growth model (EXPM) (Chowell and Viboud 2016). Intermediate values of $p$ ($0 < p < 1$) describe sub-exponential (e.g., polynomial) growth patterns. For sub-exponential growth, the closed-form solution of this equation is given by the following polynomial of degree $m$ (Tolle 2003):

$$ C\left( t \right) = \left( {\frac{r}{m}t + A} \right)^{m} $$

where $m$ is a positive integer, $A = C(0)^{1/m}$, and the deceleration parameter is given by $p = 1 - 1/m$ (Tolle 2003). An equivalent formulation of the GGM model is given by (Chowell et al. 2016b):

$$ C^{{\prime }} \left( t \right) = \mu^{{\prime }} \left( t \right)C\left( t \right) $$

where $$ \mu^{{\prime }} \left( t \right) = \left\{ {\begin{array}{*{20}l} {\frac{r}{{r\left( {1 - p} \right)t + e^{{{ \log }\left( {C\left( 0 \right)} \right)\left( {1 - p} \right)}} }}} \hfill & {0 \le p < 1} \hfill \\ r \hfill & {p = 1} \hfill \\ \end{array} } \right. $$

#### The Generalized-Richards Model (GRM)

While the GGM model characterizes early epidemic growth dynamics, the generalized-Richards model (GRM) is a useful tool to generate short-term forecasts past the peak of the 2001 foot-and-mouth disease epidemic (Chowell et al. 2016a; Pell et al. 2016). This four-parameter model is given by:

$$ C^{{\prime }} = rC^{p} \left( {1 - \left( {\frac{C}{K}} \right)^{a} } \right) $$

The GRM is an enhanced version of the standard Richards model (Wang et al. 2012) as it incorporates generalized-growth dynamics (Viboud et al. 2016). Specifically, the GRM incorporates the deceleration of growth parameter $p$ to model a range of early epidemic growth profiles ranging from constant incidence ($p = 0$), polynomial (i.e., sub-exponential) ($0 < p < 1$) and exponential growth dynamics ($p = 1$). The remaining model parameters are as follows: $r$ is the growth rate, $K$ is the final epidemic size, and parameter $a$ modulates the timing of the peak of the epidemic. The GRM model has been recently employed to generate short-term forecasts of Zika and Ebola epidemics (Chowell et al. 2016a; Pell et al. 2016).

### Parameter Estimation

We estimated model parameters for the GGM and GRM as in prior studies (Chowell 2017; Pell et al. 2016; Viboud et al. 2016). Briefly, a nonlinear least-squares fitting procedure was applied to the daily curve of notifications of infected premises during the 2001 foot-and-mouth disease epidemic in the UK. The initial number of cases $C\left( 0 \right)$ was fixed according to the first observation in the data ($C\left( 0 \right) = 1$). Nominal 95% confidence intervals for the model parameter estimates were constructed by simulating 200 realizations of the best-fit curve $C^{{\prime }} \left( t \right)$ using parametric bootstrapping with a Poisson error structure as described in Chowell et al. (2006a) and Viboud et al. (2016).

### Short-Term Forecasts

**Figure 2:**
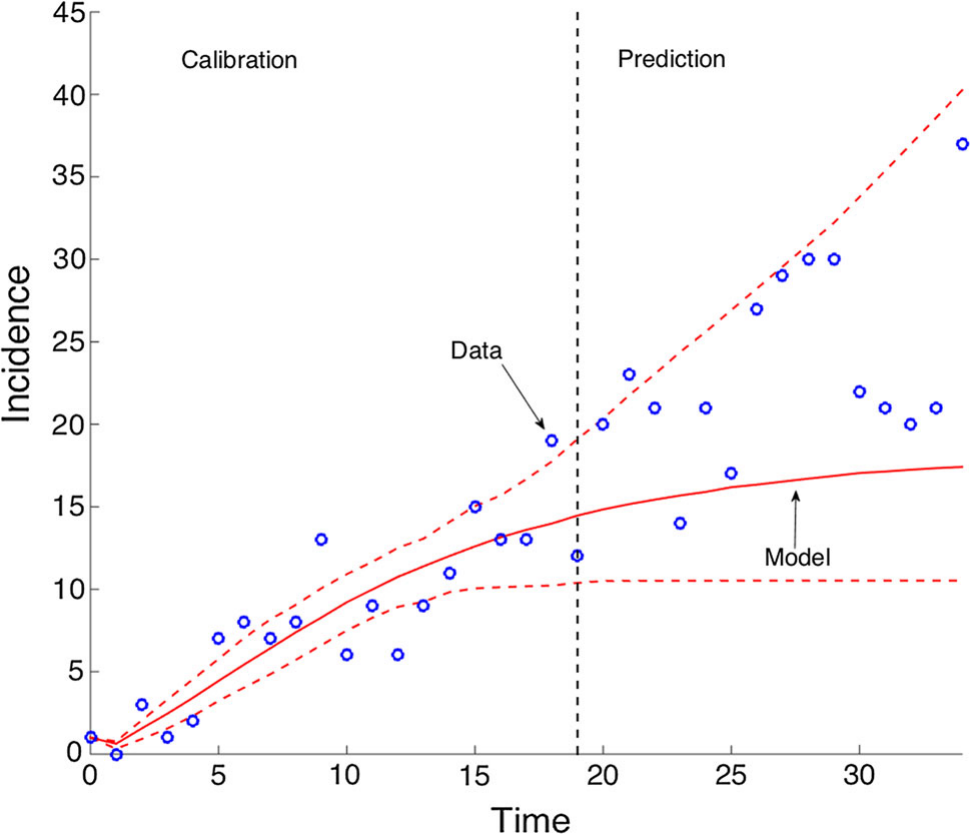
Graphic illustration of short-term forecasts provided by an epidemic model. Our short-term forecasts of the epidemic comprise a few generation intervals (prediction interval) immediately following a model-training period to estimate parameters (calibration interval).

We used simulation methods to generate an ensemble of epidemic trajectories for the short-term forecasts directly from the uncertainty of the parameter estimates (Chowell 2017; Chowell et al. 2016a; Pell et al. 2016). An illustration of model calibration and forecasting is given in Fig. 2. The performance of the models was assessed using root-mean-square error (RMSE) during the calibration and forecasting periods. We also compared the forecasting performance of the GGM and GRM models with that provided by the EXPM.

## Results

**Figure 3:**
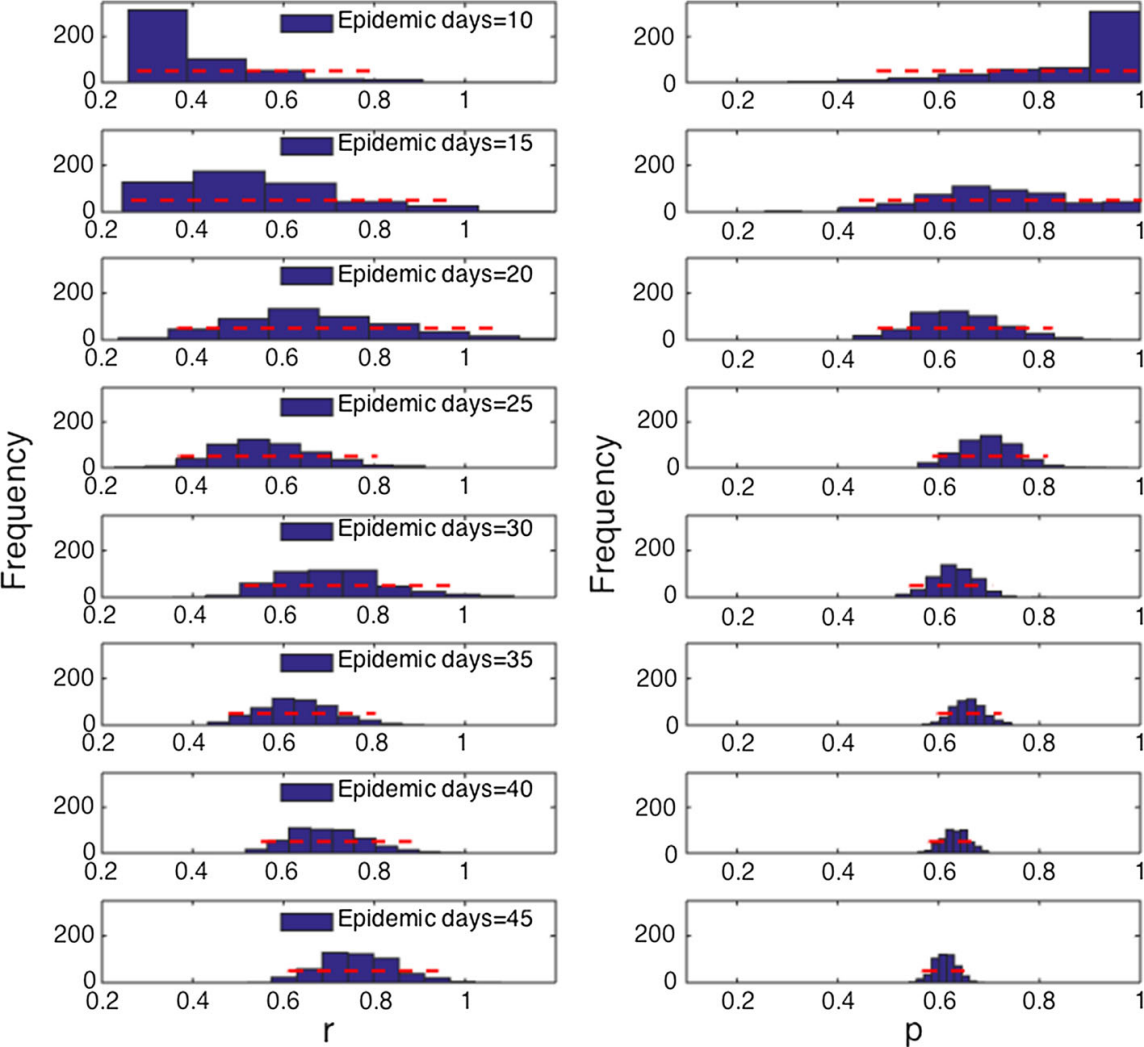
Empirical distributions (histograms) and 95% confidence intervals (red horizontal lines) for parameters $r$ and $p$ obtained by nonlinear least-square fitting the generalized-growth model (GGM) to an increasing amount of incident notification data (10–45 epidemic days). Estimates of the deceleration of growth parameter (and their uncertainty) rapidly declined as the GGM was fitted to increasing amounts of data (*ρ* = − 0.85, *P* < 0.001).

The trajectory of the 2001 FMD epidemic in the UK displays a single peak on March 21, 2001 (epidemic day 43), with most cases concentrated during February–April 2001. It is also characterized by a long “tail” with an average of three case reports per day during the last 140 days of the epidemic (Fig. 1a). The epidemic exhibits a growth-decelerating pattern as the relative growth rate declines inversely with time (Fig. 1b). The uncertainty of the parameter estimates $(r{\text{ and }}p)$ narrows down and become more precise using an increasing amount of data of the epidemic growth phase. Indeed, using only the first 10–15 days of the epidemic, the scaling of growth parameter ($p$) displays wide uncertainty (Fig. 3). The confidence interval for $p$ spans values from ~ 0.5 to 1.0, indicating 10 epidemic days of data are not sufficient to discriminate between sub-exponential (i.e., *p* < 1) and exponential growth dynamics (i.e., *p* = 1). In contrast, using 20, 25, or 30 days of epidemic data, it is possible to recover estimates of $p$ around 0.6 and the confidence interval is substantially below the exponential growth regime (Fig. 3).

**Figure 4:**
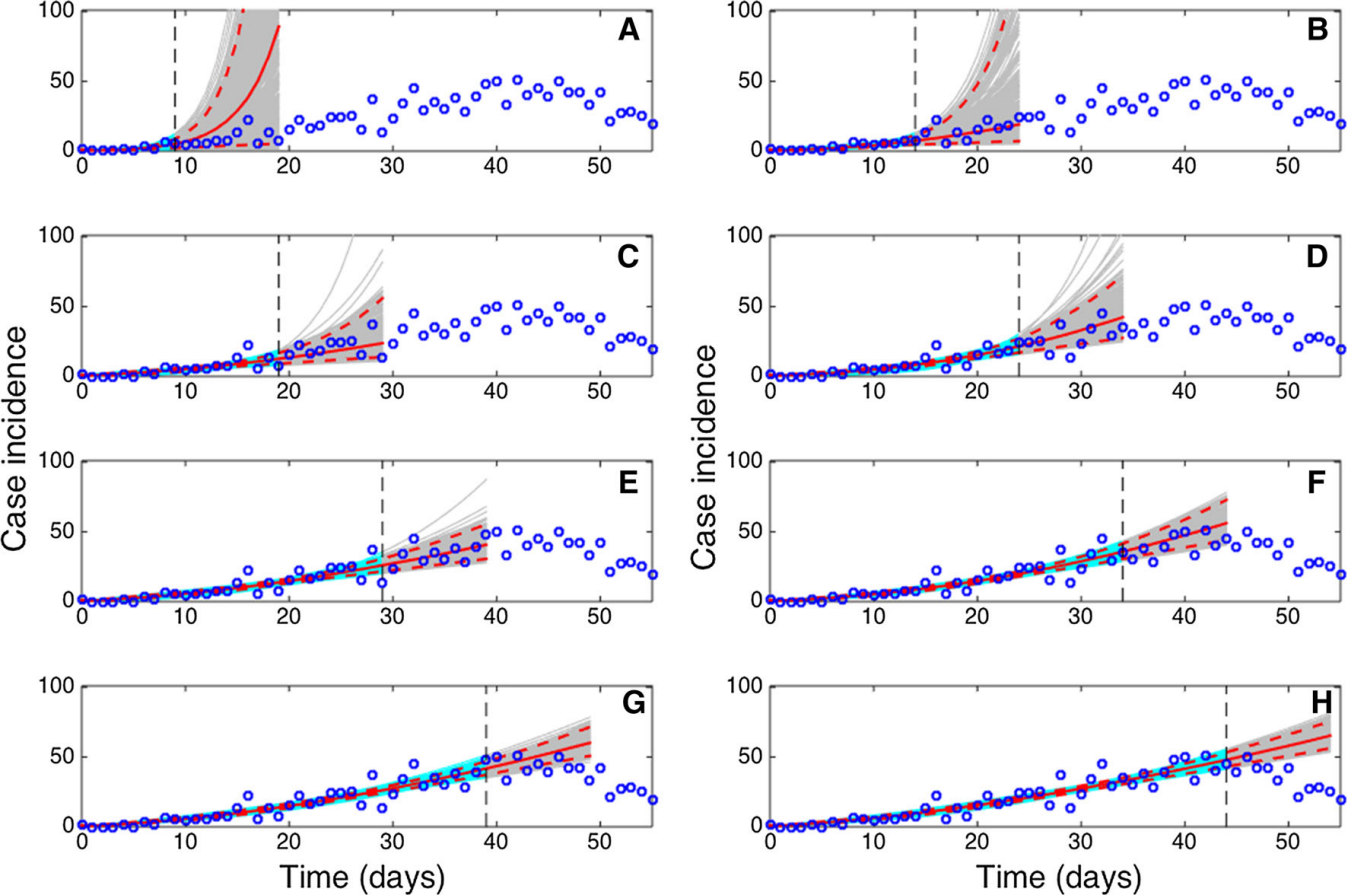
Ten-day ahead forecasts provided by the generalized-growth model (GGM) when the model is fitted to an increasing amount of epidemic data: **a** 10, **b** 15, **c** 20, **d** 25, **e** 30, **f** 35, **g** 40, and **h** 45 epidemic days. The cyan curves correspond to the uncertainty during the model calibration period, while the gray curves correspond to the ensemble of realizations for the model forecast. The mean (solid red line) and 95% CIs (dashed red lines) of the model fit ensembles (gray curves) are also shown. The vertical line separates the calibration and forecasting periods (Color figure online).

**Figure 5:**
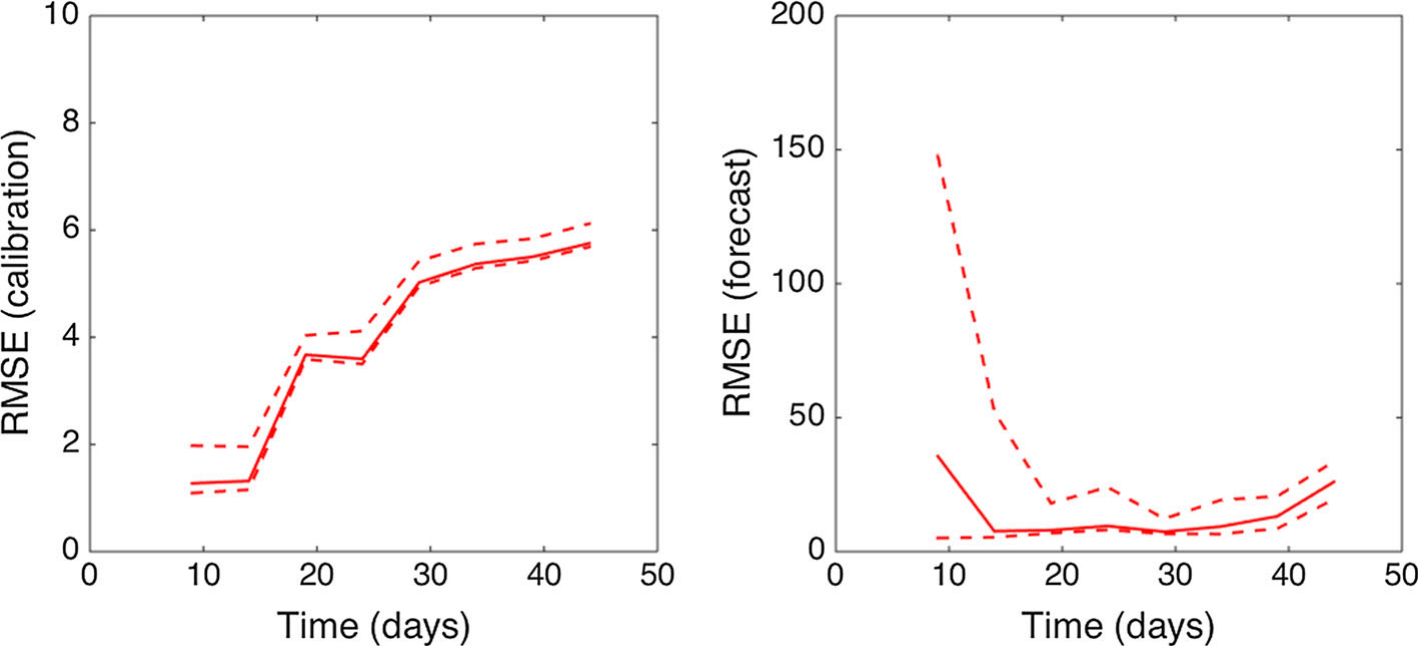
Root-mean-squared errors (RMSE) during the calibration and forecasting intervals using the generalized-growth model (GGM) when the model is fitted to an increasing amount of epidemic data: 10, 15, 20, 25, 30, 35, 40, and 45 epidemic days. The mean (solid red line) and 95% CIs (dashed red lines) of the RMSE derived from the ensemble curves are shown (see Fig. 4 for the corresponding short-term forecasts) (Color figure online).

**Figure 6:**
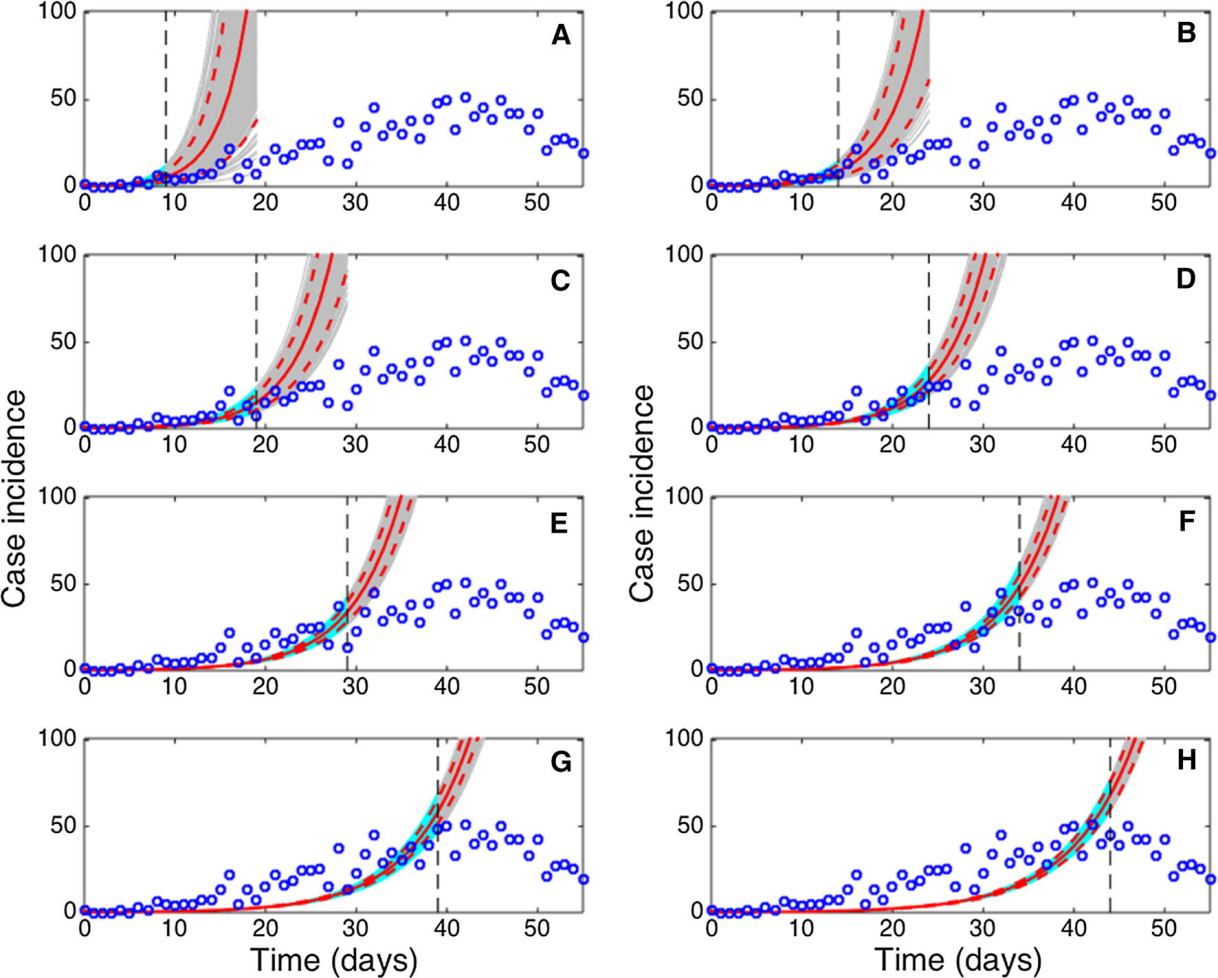
Ten-day ahead forecasts provided by the exponential growth model (EXPM) when the model is fitted to an increasing amount of epidemic data: **a** 10, **b** 15, **c** 20, **d** 25, **e** 30, **f** 35, **g** (40), and **h** 45 epidemic days. The cyan curves correspond to the uncertainty during the model calibration period, while the gray curves correspond to the ensemble of realizations for the model forecast. The mean (solid red line) and 95% CIs (dashed red lines) of the model fit ensembles (gray curves) are also shown. The vertical line separates the calibration and forecasting periods (Color figure online).

The GGM provided useful 10-day forecasts of the epidemic growth phase (Fig. 4). These forecasts faithfully tracked the ascending trajectory of the epidemic past the first 10 epidemic days (Fig. 4), with the forecast error improving and stabilizing past the first 10 epidemic days (Fig. 5). In contrast, the exponential growth model performed progressively worse (Fig. 6). The corresponding predictions of cumulative incidence derived from our 10-day ahead forecasts using an increasing amount of epidemic data of the epidemic growth phase included the target cumulative incidence, and short-term forecasts improved as the model was calibrated with an increasing length of the epidemic growth phase (not shown).

**Figure 7:**
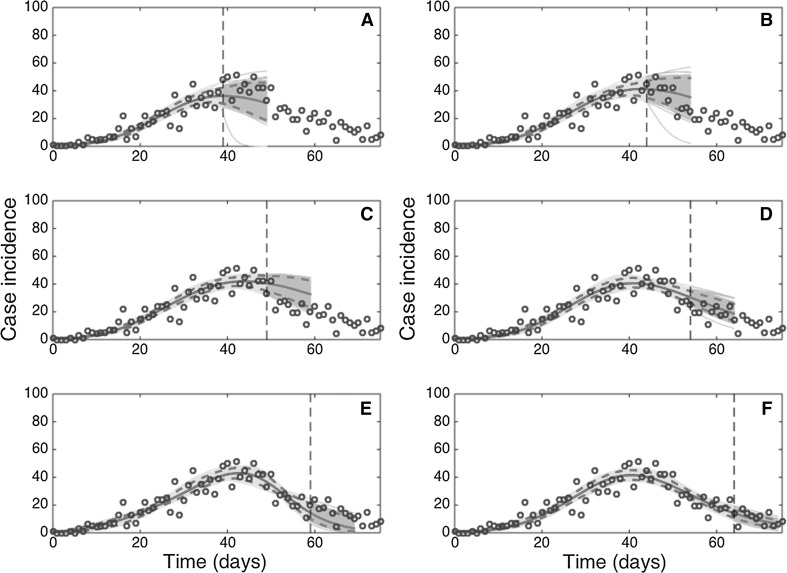
Ten-day ahead forecasts provided by the generalized-Richards model (GRM) when the model is fitted to an increasing amount of epidemic data: **a** 40, **b** 45, **c** 50, **d** 55, **e** 60, and **f** 65 days. The cyan curves correspond to the uncertainty during the model calibration period, while the gray curves correspond to the ensemble of realizations for the model forecast. The mean (solid red line) and 95% CIs (dashed red lines) of the model fit ensembles (gray curves) are also shown. The vertical line separates the calibration and forecasting periods (Color figure online).

**Figure 8:**
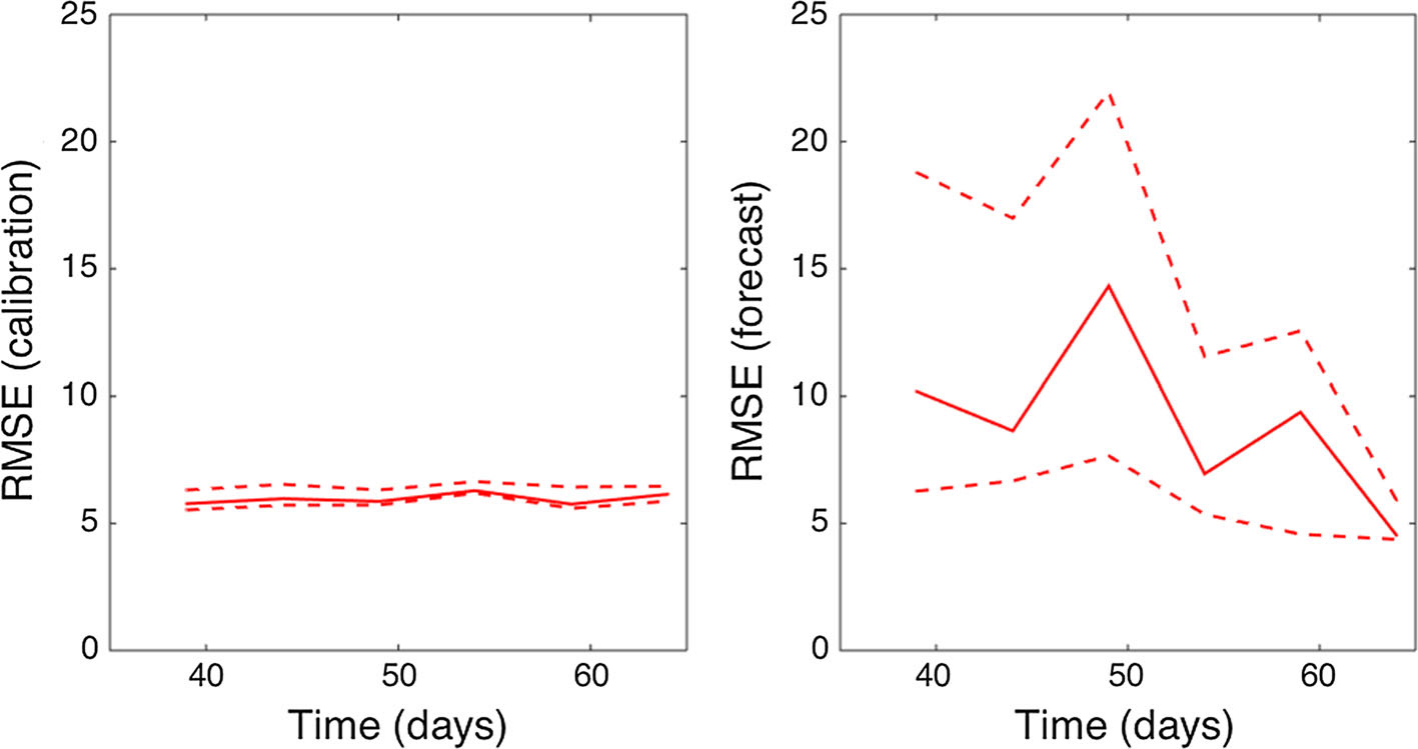
Root-mean-squared errors (RMSE) during the calibration and forecasting intervals using the generalized-Richards model (GRM) when the model is fitted to an increasing amount of epidemic data: 40, 45, 50, 55, 60, 65 days. The mean (solid red line) and 95% CIs (dashed red lines) of the RMSE derived from the ensemble curves are shown (see Fig. 7 for the corresponding short-term forecasts) (Color figure online).

Like the GGM, the GRM provided reliable 10-day ahead forecasts of the epidemic near and past the epidemic peak (Fig. 7). The forecasting error and its uncertainty declined when the GRM model was fitted to data of the epidemic past the epidemic peak (Figs. 7, 8), though the model was not able to capture the entire long tail of the FMD epidemic, particularly after day 70 of the epidemic.

## Discussion

Using a generalized-growth model (GGM), we show that the inclusion of additional data (epidemic days) significantly enhances the precision of short-term forecasts of the 2001 FMD epidemic in the UK—from high growth uncertainty that does not discriminate between exponential and sub-exponential growth to sub-exponential growth dynamics with a scaling of growth parameter substantially below 1.0 (i.e., exponential growth) (Fig. 5). The initial spread of the epidemic was characterized by undetected spread locally and via the transportation of infected sheep to markets (Gibbens et al. 2001). While this initial “seeding” of disease has been associated with the magnitude of the epidemic (Gibbens et al. 2001; Kao 2002; Kiss et al. 2006), local and national bans on the movement of livestock limited the long-distance transport of infected animals (National Audit Office 2002). Further, a nationwide cull of infected and contiguous premises was implemented about a month after the first confirmed case of infection (National Audit Office 2002; Tildesley et al. 2009). Both of these interventions likely contributed to decelerating the trajectory of the epidemic. The former was intended to limit long-distance spatial spread of the epidemic, while the latter was intended to limit local spread of the disease, e.g., animal-to-animal contact on adjacent farms and aerosol dispersal (Alexandersen et al. 2003). Culling infected and “at-risk” farms removed infectious and potentially infectious individuals; preemptive culling decreased the number of susceptible animals surrounding an infectious premise.

The initial growth in case incidence rates was due to local spread from the initial seeding of the disease—the effect of the bans on livestock movement is delayed. However, the data rapidly converge from high growth uncertainty that does not distinguish between polynomial and exponential growth toward a clear sub-exponential growth pattern (Figs. 4, 5). For comparison, the 2001 FMD epidemic in Uruguay displayed a slower early growth profile comprising 11 epidemic days with $p = 0.42$ (95% CI 0.27, 0.58) (Chowell et al. 2006b; Rivas et al. 2006; Viboud et al. 2016). The growth pattern of the FMD epidemic in the UK results from a combination of factors ranging from biological mechanisms (e.g., spatial heterogeneity in transmission dynamics), control interventions (e.g., restriction of livestock movements that limited the long-distance transport of the disease), and the incentives to farmers created by compensation schemes (e.g., the incentive effect of limiting compensation to losses on culled premises). In the absence of these bans (e.g., local and long-distance spread of the virus), case incidence rates have been hypothesized to continue to rise (Shigesada and Kawasaki 1997). Indeed, due to restrictions on animal movement, the disease was kept out of important dairying and pig industries in parts of Anglia, Midlands, southern England, western Wales, and central and northern Scotland (National Audit Office 2002). Implementation of the national cull reduced local spread of the epidemic (Keeling et al. 2001; Tildesley et al. 2009), further contributing to the overall epidemic growth pattern (Fig. 5).

In the context of highly susceptible host populations, control interventions including movement restrictions and animal culling strategies as well as spatial heterogeneity in susceptibility and infectivity in the underlying host populations are expected to shape the observed polynomial epidemic growth profile. Regardless of the specific mechanisms that explain the dynamics of epidemic growth (Chowell et al. 2015; Viboud et al. 2016), our findings emphasize the importance of forecasting approaches that do not require knowing a priori what particular factors shaped the epidemic growth profile. Specifically for FMD, there exists spatial heterogeneity in contact rates, susceptible populations of animals, and management across the UK, and different species experience different rates of susceptibility, transmission, and recovery (Alexandersen et al. 2003). Empirical epidemiological models such as the ones employed in this paper provide a tool for tracking and predicting the progression of an epidemic without explicitly modeling the mechanisms behind the particular dynamics of spread and control.

Considering only the first several weeks of an epidemic can yield much uncertainty in epidemic predictions, particularly if interventions have yet to take effect. In our model, the uncertainty in the scaling of growth parameter ($p$) using little data of the epidemic growth phase (< 16 epidemic days) did not discriminate between sub-exponential and exponential growth dynamics. At the same time, this period of the epidemic did not take into account the delayed effect of local and national livestock movement bans and livestock culling. Using an additional week of data provides improved estimates of the parameters and more accurate forecasts of the epidemic. While it is important to maintain an accurate understanding of the progression of an epidemic, scientists must take into account delays in the effects of policy interventions. Furthermore, our study suggests that in the absence of reliable information about transmission and the effects of control interventions during an emerging infection, phenomenological growth models such as the ones employed here are useful to generate short-term forecasts of epidemic growth in near real time. To be useful the method naturally requires the availability of timely case reporting so that the advantage of projecting into the near future will be worthwhile.

## Footnotes

1 In contrast, the 1967 UK and 2001 Uruguay epidemics were discovered earlier in the epidemic process and involved only bovines (Haydon et al. 2004). While other factors likely contributed more to the spread of the 2001 UK epidemic (Haydon et al. 2004; National Audit Office 2002), interspecies differences in susceptibility, transmission, and symptoms make diagnosis and management more difficult than an epidemic of a single species (Alexandersen et al. 2003).

2 The primary reason to cull infected and potentially infected animals is to minimize the risk of carrier animals. There is the potential that a recovered animal will exhibit a carrier state post-infection, though this is more common in ruminant species (Alexandersen et al. 2003). While vaccination is practiced in many parts of the world, exports of vaccination-treated products fetch lower market prices than those in which vaccination is not practiced (Knight-Jones and Rushton 2013).
